# Medicine Men of the World

**Published:** 1887-12-10

**Authors:** 


### Medicine Men of the World.

By the Man in the Crowd.

### V.—SAVAGE AND CIVILISED PRACTITIONERS.

(*Continued from p.* 155.)

In most countries it is not by any means easy to get into the medical profession, and, as has been said in a previous article, the natural consequence is, that when once the dignity has been attained, most countries hold their medicine men in the highest esteem, partly from honest belief in their powers and a sense of dependence on their good offices in times of trouble, and partly from a superstitious dread of their occult power.

Catlin, the well-known writer on the North American Indians, who spent many years among them, gives a very graphic and interesting account of "the faculty" in the backwoods. Their first prescriptions, he tells us, consist of roots and herbs, of which they have a great variety of species. When these have failed to cure, their last resource is "medicine," or mystery, for which purpose each one of them has a strange and unaccountable dress, conjured up and constructed, during a lifetime of practice, in the wildest fancy imaginable, in which he arrays himself to pay his last visit to his dying patient, over whom he dances, striking his frightful rattles, and singing songs of incantation, in the hope of curing him by charm. "There are," continues Catlin, "some instances, of course, when the enchanted unaccountably recovers, under the application of these absurd forms; and in such cases this ingenious Indian son of Esculapius will be seen for several days after on the top of a wigwam, with his right arm extended and waving over the gaping multitude, to whom he is vaunting forth, without modesty, the surprising skill he has acquired in his art, and the undoubted efficacy of his medicine or mystery." If, on the other hand, the patient dies, he soon changes his dress, and joins in doleful lamentations with the mourners, whom he assures that it was the will of the Great Spirit that the patient should die, and that any efforts he could make must therefore necessarily be unavailing.

Very impressive is this writer's account of a performance of this kind he once witnessed over a man who had had two bullets through his body, and was lying at the point of death. Many hundreds of redskins were assembled around. They were required to form a ring some thirty or forty feet in diameter, and as the apparition of the mystery man appeared in sight an awestricken "Hush—sh" went through the assembly. Then followed a dead silence, broken only by the slight tinkling of the rattles upon the great man's dress. "His *entrée* and his garb were somewhat like this: He approached the ring in a crouching position, with a slow and tilting step; his body and head were entirely covered with the skin of a yellow bear, the head of which (his own head being inside of it) served as a mask; the huge claws of which also were dangling on his wrists and ankles. In one hand he shook a frightful rattle, and in the other brandished his medicine spear or magic wand, to the rattling din and discord of all of which he added the wild and startling jumps and yelps of the Indian, and the horrid and appalling grunts and snarls and growls of the grizzly bear, in ejaculatory and guttural incantations to the good and bad spirits in behalf of his patient, who was rolling and groaning in the agonies of death, whilst the medicine man was dancing around him, jumping over him, pawing him about, and rolling him in every direction. In this wise the strange operation proceeded for half an hour before a numerous and death-like silent audience, until the man died, and the medicine man danced off to his quarters, packed up and tied and secured from the sight of the world his mystery dress and equipments."

Such concludes Catlin, is the medicine man or physician, and such is one of his wild and ridiculous manœuvres. "These men are valued as dignitaries of the tribe, and the greatest respect is paid to them by the whole community, not only for their skill in their materia medica, but more especially for their tact in magic and mysteries. They are looked upon by all as oracles of the nation. In all councils of war and peace they have a seat with the chiefs, are regularly consulted before any public step is taken, and the greatest deference and respect are paid to their opinions."

In the old world the medicine man is equally venerated by all barbarous and semi-barbarous people. We good folks who hold ourselves in the highest possible esteem for our civilisation, do not, it is true, rank our medicine men quite so high. However distinguished their services or exalted their character, we never allow them to take their seats among our great chiefs, though, perhaps, we may give them an inferior title. But in more primitive states of society the medicine man is usually a person of first-rate importance. Among the nomad Tartars, for instance, this is invariably the case. Among the Bakmains of Africa we have seen in a previous article that the profession is hereditary. With the Tartars this is not so. Only those whom the greatest authorities in the Tartar medical world recognise as 'specially qualified are eligible for the exalted rank of "Shammon." A nervous organisation is said to be indispensable, and a liability to epileptic fits constitutes an especial qualification. Perhaps persons so distinguished are less common among wandering barbarians than among ourselves, where nervous disorders are very commonly believed to have developed almost *pari passu* with social progress. A f t, everybody knows, was formerly held in this and other lands to be neither more nor less than "possession," and some such idea evidently prevailed among the Tartars. A youth subject to epileptic seizure is taken by the chief medicine man and put through a long course of training, which includes the swallowing of certain drugs and herbs. He learns to play on a sort of tambourine, and to talk in the jargon of the cult, and he must spend a whole year apart by himself in the woods, during which time it is of course not improbable that a young man picked out for his nervous excitability, and dosed with herbs calculated to produce spiritual elation, will see visions and hear voices. This will make him "Kitchi-Kama," and he is well on his way to become a fully-qualified practitioner. By the end of another twelvemonth he will be out of his novitiate, and will now become a full-blown medicine man, entitled to put up his brass plate—or whatever corresponds to it in Tartary—and to take fees.

It is usually a long and difficult process to get into the medical profession among savages; it is sometimes impossible to get out of it. Major Forbes tells of an unlucky professor of the various arts which go to make up the profession of medicine men in Ceylon, who wished to retire from business. Among other ways of turning an honest penny—honest at least as things went, no doubt, in his part of the world—the professor had been wont to make rain when required to do so. He now wished to give up this function, but his people would not hear of his doing so. They were indignant at the idea, and decreed that if he did not continue to perform his functions, in due course he should be lacerated with thorns or beaten. In his eagerness to resign his unenviable post, he frankly confessed that it was all a hoax, and that he really had no power over Jupiter Pluvius. Of course they refused to believe any such nonsense, and the chief of the district gave him imperative orders to be off, and make rain at a certain village where it was greatly needed. As he still protested that his art was only deception and imposture, he was soundly thrashed, and but for the intervention of Major Forbes, it seemed probable that the poor old man would have backed out of his profession under the cudgels of those who insisted on his sticking to it.

(*To be continued.*)

